# Arrhythmic risk stratification in arrhythmogenic right ventricular cardiomyopathy

**DOI:** 10.1093/europace/euad312

**Published:** 2023-11-03

**Authors:** Alessio Gasperetti, Cynthia A James, Richard T Carrick, Alexandros Protonotarios, Anneline S J M te Riele, Julia Cadrin-Tourigny, Paolo Compagnucci, Firat Duru, Peter van Tintelen, Perry M Elliot, Hugh Calkins

**Affiliations:** Division of Cardiology, Department of Medicine, Johns Hopkins University, Blalock 545, 600 N. Wolfe St., Baltimore, MD 21287, USA; Department of Genetics, University Medical Center Utrecht, University of Utrecht, Heidelberglaan 100, Utrecht, The Netherlands; Department of Medicine, Division of Cardiology, University Medical Center Utrecht, Utrecht University, Heidelberglaan 100, Utrecht, Utrecht, The Netherlands; Division of Cardiology, Department of Medicine, Johns Hopkins University, Blalock 545, 600 N. Wolfe St., Baltimore, MD 21287, USA; Division of Cardiology, Department of Medicine, Johns Hopkins University, Blalock 545, 600 N. Wolfe St., Baltimore, MD 21287, USA; Department of Cardiology, UCL Institute of Cardiovascular Science, London, UK; Department of Medicine, Division of Cardiology, University Medical Center Utrecht, Utrecht University, Heidelberglaan 100, Utrecht, Utrecht, The Netherlands; Cardiovascular Genetics Center, Montreal Heart Institute, Université de Montréal, Montréal, QC, Canada; Cardiology and Arrhythmology Clinic, Marche University Hospital, Ancona, Italy; Department of Cardiology, Arrhythmia Unit, University Heart Center, University Hospital Zurich, Zurich, Switzerland; Department of Genetics, University Medical Center Utrecht, University of Utrecht, Heidelberglaan 100, Utrecht, The Netherlands; Department of Cardiology, UCL Institute of Cardiovascular Science, London, UK; Division of Cardiology, Department of Medicine, Johns Hopkins University, Blalock 545, 600 N. Wolfe St., Baltimore, MD 21287, USA

**Keywords:** Arrhythmogenic right ventricular cardiomyopathy, Risk stratification, Primary Prevention, ICD

## Abstract

Arrhythmogenic right ventricular cardiomyopathy (ARVC) is a heritable cardiomyopathy characterized by a predominantly arrhythmic presentation. It represents the leading cause of sudden cardiac death (SCD) among athletes and poses a significant morbidity threat in the general population. As a causative treatment for ARVC is still not available, the placement of an implantable cardioverter defibrillator represents the current cornerstone for SCD prevention in this setting. Thanks to international ARVC-dedicated efforts, significant steps have been achieved in recent years towards an individualized, patient-centred risk stratification approach. A novel risk calculator algorithm estimating the 5-year risk of arrhythmias of patients with ARVC has been introduced in clinical practice and subsequently validated. The purpose of this article is to summarize the body of evidence that has allowed the development of this tool and to discuss the best way to implement its use in the care of an individual patient.

## Introduction

Arrhythmogenic right ventricular cardiomyopathy (ARVC) is a heritable cardiomyopathy characterized by a predominantly arrhythmic presentation out of proportion to the underlying structural disease and with the histological hallmark of scarring and/or fibro-fatty infiltration of the ventricular myocardium.^1–4^ Arrhythmogenic right ventricular cardiomyopathy is the most studied and best-characterized disease within the phenotypic spectrum of arrhythmogenic cardiomyopathy (ACM), and numerous different underlying genes have been identified, which, in the presence of disease-causing variants, lead to the development of ARVC, as summarized in *Table 1*. Regardless of the underlying genetic basis, all forms of ARVC are associated with an increased risk of sustained ventricular arrhythmias (VAs) and sudden cardiac death (SCD).^8^ It is notable that ARVC is 10 times less common than hypertrophic cardiomyopathy but results in a higher proportion of unexplained cardiac deaths in autopsy series, and it is one of the most common causes of SCD among athletes.^1,2,9,10^

**Table 1:** Genes associated with ARVC^5^

	Localization	Inheritance	Phenotype	Peculiarities	Dedicated risk stratification?
Plakophillin 2 (*PKP2*)	Desmosome	Ad	Right dominant	Highest susceptibility to exercise	No but prototype for ARVC risk calculator^6^
Desmoplakin (*DSP*)	Desmosome	Ad/AR	Biventricular or left ventricular	Hair and skin features	No
				Myocarditis-like episodes	
Desmoglein 2 (*DSG2*)	Desmosome	Ad/AR	Biventricular		No
Desmocollin 2 (*DSC2*)	Desmosome	Ad/AR	Right dominant		No
Junction plakoglobin (*JUP*)	Desmosome	AR	Right dominant or biventricular	Hair and skin features	No
				Naxos disease	
Desmin (*DES*)	Intermediate filament	Ad	Right dominant	AV conduction disorders	No
				Skeletal myopathies possible	
Transmembrane protein 43 (*TMEM43*)	Nuclear envelope	Ad	Biventricular or left ventricular	High risk of VA	No
				Male	
Phospholamban (*PLN*)	Calcium handling	Ad	Biventricular or left ventricular		Yes^7^

Once a diagnosis of ARVC is established,^11^ the next step in management is to assess an individual’s risk of VA/SCD and determine whether the placement of an implantable cardioverter defibrillator (ICD) is recommended, especially when dealing with patients without previous VA events (the so-called primary prevention ARVC patients).^12^ The purpose of this review article is to summarize the large body of evidence that has allowed the development of modern tools for risk stratification in patients with ARVC and the best way to implement its use in the care of an individual patient.

## Patient management and arrhythmic risk stratification

The cornerstone of SCD prevention in patients with ARVC is the placement of an ICD.^13^ However, in a young and active population such as the one affected by ARVC, the potential absolute risk of SCD reduction achieved with ICDs should be carefully weighed against the risk of device-related complications. Multiple studies have shown that both transvenous and subcutaneous ICDs are associated with complications,^14–17^ with a meta-analysis showing a potential 3.9% pooled risk annual rate of inappropriate shocks and a 4.2% annual rate of other complications, such as infection or lead malfunction for young patients implanted with an ICD for the management of familial cardiomyopathies.^18^ Performing an accurate risk-benefit analysis of ICD implantations in patients with ARVC is therefore a critical part of the integrative management of these patients.

### Known predictors and current guidelines

**Figure 1:**
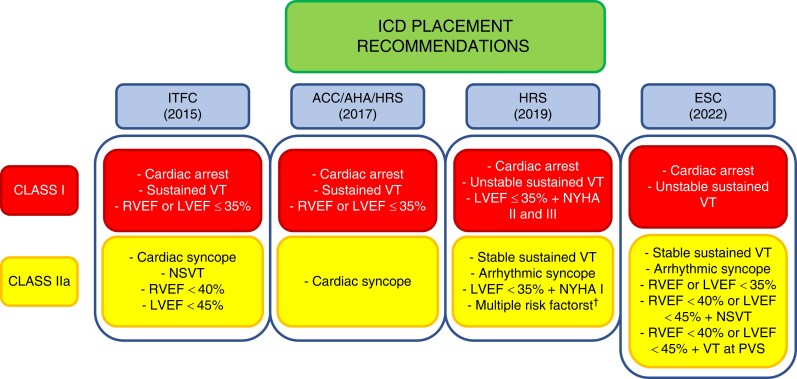
Summary of current guideline indications for ICD placement in patients with ARVC.

Numerous studies have reported associations between demographic, clinical, and genetic characteristics and the development of sustained VAs in patients with ARVC (*Table 2*). These include young age and male sex, and it has been speculated that this results from the pro-arrhythmic effects of testosterone and other sex hormones.^42,43^ Findings from 12-lead electrocardiograms (ECGs) (i.e. number of T-wave inversions (TWIs) and QRS complex fractionation), 24-h ambulatory ECG monitoring [i.e. premature ventricular contraction (PVC) burden, PVC spikes, and non-sustained ventricular tachycardia (NSVT)], and cardiac imaging [i.e. right ventricular (RV) and left ventricular (LV) dysfunction] have also been identified as important predictors of arrhythmic risk.^6,20,30,31,34,35,38,41,44–47^ Additionally, the results of invasive electrophysiological tests including inducibility of VT during programmed ventricular stimulation (PVS) or the presence of low voltage areas or areas of fractioned potentials on electro-anatomical mapping may have predictive value in some ARVC cohorts.^33,36,37^ By combining these risk markers and the presence of previous sustained arrhythmic events, the 2015 International Task Force (ITFC) consensus for the treatment of ARVC, the 2017 American College of Cardiology (ACC)/American Heart Association (AHA)/Heart Rhythm Society (HRS) guidelines for management of patients with VAs, the 2019 HRS consensus document on ACM, and the 2022 European Society of Cardiology (ESC) guidelines for the management of patients with VAs have provided expert recommendations on how to risk stratify for ICD placement in patients with ARVC^13,48–50^ (*Figure 1*). These guidelines have subsequently been compared by Bosman *et al*.^51^ Regardless, all above-mentioned guideline recommendations were based on expert opinion, only provided crude estimates of risk (e.g. <1%/year or 1–10%/year), and did not take into account potentially correlated risk factors. A more personalized and direct approach to risk assessment was therefore desired.

**Table 2:** Predictors at baseline of sustained ventricular arrhythmic events (modified and integrated from Krahn *et al*.^19^)

First author/year	*N* of patients	Predictor	OR/HR
Age			
Orgeron (2017)^20^	312	Age < 30	3.14
Cadrin-Tourigny (2019)^6^	528	Age (1-year increase)	0.98
Cadrin-Tourigny (2021)^21^	864	Age (1-year increase)	0.96
Carrick (2022)^22^	408	Age (1-year increase)	0.978
Sex			
Mazzanti (2016)^23^	301	Male	2.49
Martin (2016)^24^	26	Male	1.60
Lin (2017)^25^	70	Male	2.41
Cadrin-Tourigny (2019)^6^	528	Male	1.63
Cadrin-Tourigny (2021)^21^	864	Male	1.99
Carrick (2022)^22^	408	Male	1.746
Protonotarios (2022)^26^	554	Male	1.734
Exercise			
Mazzanti (2016)^23^	301	Exercise	2.98
Bosman (2022)^27^	178	Exercise > 30 METh/week	3.00
Cardiac syncope			
Corrado (2010)^28^	106	Syncope	2.94
Battipaglia (2012)^29^	30	Unexplained syncope	16.1
Mazzanti (2016)^23^	301	Syncope	3.36
Cadrin-Tourigny (2019)^6^	528	Cardiac syncope < 6 m.o.	1.93
Carrick (2022)^22^	408	Cardiac syncope < 6 m.o.	1.554
Protonotarios (2022)^26^	554	Cardiac syncope < 6 m.o.	2.672
QRS			
Canpolat (2013)^30^	78	QRS interval fractionation	6.52
T-wave inversion			
Cadrin-Tourigny (2019)^6^	528	*n* of leads with TWI	1.12
Cadrin-Tourigny (2021)^21^	864	*n* of leads with TWI	1.12
Carrick (2022)^22^	408	*n* of leads with TWI	1.10
Protonotarios (2022)^26^	554	*n* of leads with TWI	1.36
PVS			
Bhonsale (2011)^31^	84	PVS inducibility	4.50
Orgeron (2017)^20^	312	PVS inducibility	2.28
Casella (2020)^32^	101	PVS inducibility	8.9
Gasperetti (2022)^33^	288	PVS inducibility	2.52
Non-sustained VT			
Bhonsale (2011)^31^	84	Non-sustained VT	10.50
Cappelletto (2018)^34^	98	Non-sustained VT	3.28
Cadrin-Tourigny (2019)^6^	528	Non-sustained VT	2.25
Gasperetti (2022)^35^	169	Non-sustained VT	2.29
Carrick (2022)^22^	408	Non-sustained VT	2.126
Protonotarios (2022)^26^	554	Non-sustained VT	1.36
EAM derived			
Santangeli (2012)^36^	32	Fragmented potentials	21.22
Migliore (2013)^37^	69	Low voltage areas	1.70
Lin (2017)^25^	70	Low potential areas	1,07
Casella (2020)^32^	101	Late fragmented potentials	7.4
PVC			
Orgeron (2017)^20^	312	PVC burden > 1000/24 h	4.43
Orgeron (2018)^38^	365	PVC burden > 1000/24 h	5.24
Cadrin-Tourigny (2019)^6^	528	(log) 24-h PVC burden	1.19
Cadrin-Tourigny (2021)^21^	864	(log) 24-h PVC burden	1.12
Gasperetti (2022)^35^	169	(log) 24-h PVC burden	1.50
Carrick (2022)^22^	408	(log) 24-h PVC burden	1.321
Protonotarios (2022)^26^	554	(log) 24-h PVC burden	1.167
RV function			
Sarvari (2011)^39^	69	RV strain (1% decrease)	1.25
Sarvari (2011)^39^	69	RV FAC (5% decrease)	2.33
Canpolat (2013)^30^	78	RVEF reduction	3.76
Cappelletto (2018)^34^	98	RV FAC (1% increase)	0.35
Cadrin-Tourigny (2019)^6^	528	RVEF (1% decrease)	1.03
Bourfiss (2022)^40^	132	RV strain (1% decrease)	1.05
LV function			
Sarvari (2011)^39^	69	LV global longitudinal strain (1% decrease)	1.41
Canpolat (2013)^30^	78	LV involvement	2.88
Aquaro (2020)^41^	140	LV involvement	4.20
Aquaro (2020)^41^	140	LV dominant phenotype	3.40
Bourfiss (2022)^40^	132	LV strain (1% decrease)	1.22
Miscellanea			
Battipaglia (2012)^29^	30	RR variability in the LF amplitude	0.88
Mazzanti (2016)^23^	301	History of atrial fibrillation	4.38

Only studies reporting (i) a measure of association with arrhythmic events and (ii) patients with a definite diagnosis of ARVC by Task Force Criteria have been included in this table.

### The arrhythmogenic right ventricular cardiomyopathy risk calculator

While there is consensus about the benefits of ICDs in patients with ARVC who have experienced previous episodes of sustained VAs,^13,48,49^ the indications for primary prevention ICD placement in patients with ARVC and no such history remain controversial as many studies have reported poor performance of the existing approach among patients without previous VA, with a high number of ICD implanted per sustained VA treated.^6,20,51^

To better inform medical providers and patients when making the decision on whether to implant an ICD for primary prevention, a risk stratification tool that generates individualized estimates was proposed by a multinational collaboration in 2019.^6^ This tool, called the ARVC risk calculator, employs seven clinical variables [age, sex, number of leads with a negative T wave in a 12-lead ECG, 24-h PVC burden, NSVT, history of a recent (<6 months) cardiac syncope episode, and RV ejection fraction (RVEF)% from cardiac magnetic resonance] in a model that provides 5-year risk estimates for a composite outcome of sustained VTs, ventricular fibrillation/flutter, SCD, and appropriate ICD therapies. It was developed from a multicentre cohort of 528 patients from six countries who fulfilled definite 2010 Task Force Criteria for ARVC and showed a good internal reliability with a bootstrapped *C* statistic of 0.77 (0.73–0.81). A subsequent study from the same collaboration modified the risk calculator to include an estimation for the risk of rapid VA events (>250 b.p.m.).^21^ The clinical variables used in this calculator are derived from clinical tests recommended by available guidelines and are routinely collected in most ARVC/cardiomyopathy clinics. This makes the ARVC risk calculator easy to implement into clinical workflow.^13,52^ Additionally, its integrative approach results in a single numerical output that could be used for informed decision-making conversations between patients and healthcare providers. Finally, the analyses have demonstrated that ARVC risk tool risk performs better than the 2015 TFC consensus recommendations for ICD placement. Specifically, the ARVC risk calculator approach resulted in the same protection from VAs but with the advantage of a 20.3% reduction in the number of ICDs.

#### Validation of the arrhythmogenic right ventricular cardiomyopathy risk tool

Multiple independent study groups have tested the performance of the ARVC risk calculator in cohorts of patients with ARVC in Europe and Asia. These include two cohorts of 88 primary prevention^32^ and 140 mixed primary and secondary prevention ARVC patients from Italy,^41^ one study from France (115 primary prevention ARVC patients)^53^ and another from China (88 mixed primary and secondary prevention ARVC patients).^54^ All reported similar results, showing high discriminatory performance for VA of the risk calculator in those in whom the ARVC calculator was originally developed. These studies were, however, hampered by a relatively low sample size, but, in 2022, two larger independent studies were simultaneously published.^26,55^ Jordà *et al*.^55^ corroborated the effectiveness and reliability of the ARVC risk calculator, reporting good discrimination [*C* statistic: 0.70 (0.65–0.75)] in a large, multicentre cohort composed of 429 ARVC patients enrolled from 29 centres in North America and Europe. The findings derived from a cohort of 554 ARVC patients led Protonotarios *et al*.^26^ to similar conclusions [overall *C* statistic: 0.75 (0.70–0.81)]. However, this second study reported limited calibration of the model with risk overestimation across all risk strata. Furthermore, overall performance was variable between genotypes, with the best fit found within carriers of *PKP-2* disease-causing variants and more limited performance in the gene-elusive population. The most recent ESC guidelines for the management of cardiomyopathies have now endorsed the use of the ARVC risk calculator.^56^*Table 3* lists all studies of the ARVC risk calculator including its derivation, external validation, and refinement that have been currently published.

**Table 3:** Original development study, external validation studies, and additional calculator refinements

	Pts (*n*)	Pts with ICD at baseline (*n*/%)	Follow-up (years)	Total events (*n*/%)	ICD shocks (*n*/%)	Findings	Comments
Original development study							
Cadrin-Tourigny *et al*.^6^ (2019)	528	218 (41.3)	4.83 (2.44–9.33)	146 (27.7)	102 (19.3)	Overall *C* statistic: 0.77 (0.73–0.81)	Development of the ARVC risk calculator
External validation studies							
Casella *et al*.^32^ (2020)	82	54 (65.9)	5.41 (2.59–8.37)	28 (34.1)	23 (28.0)	Good performance of risk calculator in classic ARVC forms	Risk calculator underpredicts risk in BiV/LD forms
Gasperetti *et al*.^57^ (2020)	20	7 (35.0)	5.3 (3.2–6.6)	6 (30.0)	5 (25.0)	Good performance of risk calculator in ARVC patients with a high exercise exposure	Very high-end endurance athlete cohort
Aquaro *et al*.^41^ (2020)	140	51 (36.4)	5.0 (2.0–8.0)	48 (34)	33 (23.6)	Good performance of risk calculator in classic ARVC forms	Mix of primary/secondary prevention pts; risk calculator underpredicts risk in BiV/LD forms
Baudinaud *et al*.^53^ (2021)	115	1 (0.9)	7.8 (6.1–9.7)	15 (13.0)	2 (1.7)	*C* statistic: 0.84 (0.74–0.93)	Risk overestimation for low risk patients
Zhang *et al*.^54^ (2022)	88	70 (79.5)	3.9 (1.6–6.9)	57 (64.8)	57 (64.8)	Overall *C* statistic: 0.681 (0.567–0.796)	Mix of primary and secondary prevention pts
						Primary prevention *C* statistic: 0.833 (0.615–1.000)	
						Secondary prevention *C* statistic: 0.640 (0.510–0.770)	
Protonotarios *et al*.^26^ (2022)	554	263 (47.5)	6.0 (3.1–12.5)	100 (18.1)	52 (9.3)	Overall *C* statistic: 0.75 (0.70–0.81)	Significant impact of genotype on risk calculator performance
						Gene-positive *C* statistic: 0.82 (0.76–0.88)	
						Gene-elusive *C* statistic: 0.65 (0.57–0.74)	
						*PKP-2 C* statistic: 0.83 (0.75–0.91)	
						*DSP C* statistic: 0.80 (0.53–0.96)	
Jordà et al^55^ (2022)	429	175 (40.8)	5.02 (2.05–7.90)	103 (24)	61 (14.2)	*C* statistic: 0.70 (0.65–0.75)	Main validation study
Additional calculator refinements							
Bosman *et al*.^27^ (2022)	176	N/A	5.4 (2.7–9.7)	53 (30.1)	40 (22.7)	*C* statistic: 0.77 (0.71–0.84)	No need for exercise correction in the risk calculator estimates
Gasperetti *et al*.^33^ (2022)	288	78 (27.1)	5.31 (2.89–10.17)	120 (41.7)	89 (30.9)	Integrated C statistic of risk calculator + PVS: 0.75	Maximal benefit of PVS in moderate risk patients (<25% 5-year predicted risk) for ICD exclusion
Bourfiss *et al*.^40^ (2022)	132	68 (51.5)	4.3 (2.0–7.9)	25 (19.0)	22 (16.7)	C statistic Risk Calc: 0.76 (0.63–0.90)	Inclusion of CMR derived LV global and septal circumferential strain does not improve the model
						Integrated *C* statistic risk calc + LV strain: 0.82 (0.72–0.92)	

## Refinement of the arrhythmogenic right ventricular cardiomyopathy risk calculator

In the years following its development, a series of studies have aimed to improve and refine the ARVC risk calculator by assessing the role of variables that were not originally included and the impact of disease development during follow-up.^52^

###  

#### The role of physical exercise

Physical exercise is a well-known risk factor in patients with ARVC.^58,59^ Multiple studies have shown that physical exercise, and in particular endurance training, is associated with an increase in disease penetrance, arrhythmic risk, and adverse cardiovascular outcomes in patients with ARVC.^60,61^ A clear dose-response association between the quantity of physical exercise and an increase of risk has been shown,^27,60^ as well as a significant improvement in clinical parameters (RVEF, PVC burden, NSVT, and stress test response) and a decrease of VA rates after de-training and exercise restriction.^57,62^ Because of the close link between exercise and ARVC, a diagnosis of ARVC represents a contraindication to competitive sports eligibility, and patients with ARVC are recommended to limit the amount of vigorous endurance exercise they perform.^13,48,50^

In the first iteration of the ARVC risk calculator, no risk estimate correction for exercise exposure was included and it was therefore questioned whether this tool would adequately perform in ARVC patients with a high-dose exercise exposure. This question was first tested by Gasperetti *et al*. in a cohort of 20 high-end endurance athletes diagnosed with ARVC. Although underpowered, in this cohort, the ARVC risk calculator yielded a good performance, with an almost perfect overlap between predicted and observed risk.^57^ These findings were later confirmed and expanded in a larger study performed by Bosman *et al*.^27^ in which 176 definite diagnosis ARVC patients without prior sustained VA at the time of diagnosis underwent interview-based lifetime exercise exposure assessment. As expected, physical exercise at diagnosis was strongly associated with a higher arrhythmic risk in follow-up. The ARVC risk calculator performance for VA risk stratification, however, remained high [*C* statistic: 0.77 (0.71–0.84)] at all levels of exercise exposure (>18, >24, and >36 METh/week), and no significant improvement in model performance was shown when exercise exposure was included. Bosman *et al*. hypothesized that the performance of the ARVC risk calculator was maintained in athletes because high-level exercise exposure was strongly associated with at least five of the seven variables already included in the risk calculator (namely, young age, higher PVC count, more TWI at 12-lead ECG, NSVT, and lower RVEF) allowing its use in athletic and sedentary ARVC patients alike. While it is of paramount importance to recommend exercise detraining in patients with ARVC already at their first visit to reduce future events, the amount of exercise exposure does not seem to impair the performance of the risk stratification tool.

#### Advanced imaging and the arrhythmogenic right ventricular cardiomyopathy risk calculator

Several advances in cardiac imaging permit the identification of additional parameters that could be of help when performing risk stratification assessments in patients with ARVC. Late gadolinium enhancement (LGE) on cardiac magnetic resonance (CMR) assessment, representing fibrosis, has been reported as a predictor of arrhythmic events in LV cardiomyopathies,^63–65^ but LGE assessment in the RV is technically much more difficult due to the thinness of the RV wall. For this reason, data addressing the role of LGE in ARVC are limited, with most of the available studies focusing on the value of LV LGE,^41^ which is generally associated with advanced stages of disease. The relative importance of LGE presence on the risk of arrhythmic outcomes in ARVC is therefore still an understudied topic, and its potential additional role in risk stratification on top of currently available tools requires investigations.

There are more data on the relationship between speckle tracking and myocardial strain assessments and risk. Multiple reports have shown associations between reduced myocardial strain and arrhythmic outcomes in ARVC.^40,66–70^ However, the integration of these findings with standardized risk assessment strategies such as the ARVC risk calculator had not been attempted until very recently. In a recent study of 132 patients with ARVC and no prior VA events by Bourfiss *et al*.,^40^ RV and LV CMR-derived strains were shown to be significantly associated with VA events during follow-up. However, both parameters lost statistical significance after correcting for RVEF, LVEF, or the predicted arrhythmic risk derived from the ARVC risk calculator. Similarly, the performance of the ARVC risk calculator was not shown to improve significantly if the CMR-derived strain parameter with the strongest association with arrhythmic events (namely, the LV global and septal circumferential strain) was added to the model. It is important to note, however, that the study largely consisted of ARVC patients with right-dominant disease (64% were *PKP2* carriers) and may have been underpowered to evaluate strain as an arrhythmic risk predictor in those with biventricular or left-dominant disease. Additionally, it should be noted that standardization of myocardial speckle tracking is an important scientific and clinical problem and these specific findings may not be fully replicable in imagining obtained through a different imaging software.

### Programmed ventricular stimulation in primary prevention assessments

Another area of potential improvement for the ARVC risk calculator was the integration of VT inducibility during PVS. Over the years, the role of PVS for arrhythmic risk stratification or patients with ARVC has been extensively debated, with some studies reporting a poor positive predictive value^28^ and multiple others suggesting it could have a significant role in the risk stratification process.^20,31,71–74^ These studies have been hampered by small sample sizes, non-uniform PVS protocols, and the inclusion of patients with both borderline and definite diagnoses of ARVC, as well as both patients with and without a history of previous sustained VA. For these reasons, clear data addressing the utility of PVS in patients with ARVC and no previous VA events were lacking until recently.

A recent multicentre study from Gasperetti *et al*.^33^ reported data from 288 patients with definite ARVC without a previous history of sustained VA undergoing PVS. Half of the study cohorts were inducible for monomorphic VT. Inducibility was a strong independent predictor of sustained VA during follow-up above and beyond the predictions of the risk calculator. Through a Bayesian analysis, PVS inducibility was integrated into the risk predictions from the ARVC risk calculator pre-test probability, offering a refined 5-year risk estimation and improving the performance of the prediction model. The maximal benefit of PVS results was observed in patients with a low/moderate ARVC risk calculator-derived risk (5-year risk < 25%). In this subset of patients, PVS yielded a high negative predictive value (92.6%) for VA. A negative PVS result therefore can be used as an additional factor in favour of deferring ICD use. The arvcrisk.com website has been updated to allow for individual calculation using this Bayesian approach.

### Longitudinal assessment of arrhythmic risk over time

The ARVC risk calculator was developed to provide 5-year arrhythmic risk estimation and to aid the decision-making process at a single time point. ARVC, however, is a progressive condition, and patient risk profiles may change over time due to the dynamic nature of the arrhythmic substrate.^35,75,76^ Thus, initial arrhythmic risk assessments in ARVC patients may not hold true during longitudinal follow-up. Patients initially at low arrhythmic risk may move towards higher risk brackets (or vice versa), potentially benefitting from a follow-up conversation regarding the need for ICD. Additionally, transient ‘hot phases’ of active inflammation and increased arrhythmic risk have been described during the natural history of this disease.^77^ It is therefore of paramount importance to re-assess ARVC patients during follow-up.

While the impact of repeated testing and longitudinal risk stratification in ARVC is understudied, a number of recent studies provide insight into this important clinical question. In agreement with new recommendations for the repeated use of ambulatory cardiac monitoring every 12–18 months for re-assessment of arrhythmic risk in ARVC patients,^35^ changes in the burden of PVCs and NSVT have been shown parallel arrhythmic risk. In particular, sudden increases in the number of PVCs (sometimes referred to as ‘PVC spikes’) on Holter monitoring are associated with increased arrhythmic risk in the year immediately following assessment. These data were recently confirmed and integrated by Carrick *et al*.,^22^ who reported on the dynamic performance of the ARVC risk calculator during longitudinal follow-up. This decrement in predictive discrimination, however, was negated through repeat estimation of 5-year arrhythmic risk using the ARVC risk calculator and updated assessments of clinical risk factors (e.g. repeated 24-h Holter, echocardiograms, and CMRs). Incorporating these updated risk factors into repeated predictions meant that the performance of the ARVC risk calculator remained excellent during long-term follow-up [*C* statistic ranging between 0.83 (0.80–0.86) and 0.79 (0.73–0.85)]. Repeated use of the ARVC risk calculator for dynamic arrhythmic risk assessment using updated clinical risk factors seems effective and, given current expert consensus recommendations for repeated clinical examinations, may be reasonably easy to implement within the everyday workflow of ARVC clinics. Additional prospective studies on this topic are clearly needed, with the goal of supporting new, data-driven recommendations for longitudinal arrhythmic risk assessment in ARVC.

## Comparison of the arrhythmogenic right ventricular cardiomyopathy risk calculator with other guidelines

**Figure 2:**
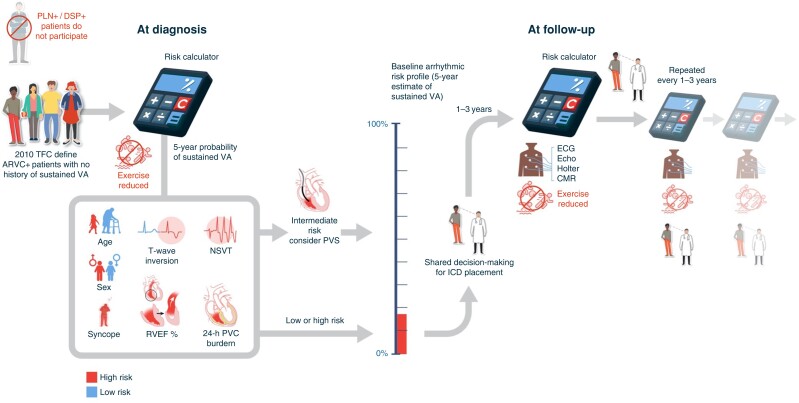
Summary of characteristics of the ARVC risk calculator and its implementation in the clinical workflow.

Current data suggest that the ARVC risk calculator is a useful adjunct to risk stratification in ARVC **(***Figure 2***).** That said, the decision of whether to use this tool in lieu of other stratification algorithms (e.g. the 2015 ITFC consensus, the 2017 AHA guidelines for SCD, or the 2019 HRS consensus) should depend on the reliability and accuracy of this tool compared to alternative strategies in the prediction of VA events. In the original publication, a hypothetical strategy for ICD decision-making based upon the ARVC risk calculator demonstrated superior clinical net benefit (defined as the number of ICDs placed for treated event) compared with the 2015 ITFC Consensus regardless of the threshold used for recommending ICD implantation. There, the same level of protection from VA events was achieved with an average 20.3% reduction in ICD implantation.^6^ A subsequent analysis from Aquaro *et al*.^78^ showed an ARVC risk calculator 5-year estimated risk threshold of 10% for ICD implant achieving a higher protection rate and clinical net benefit than both 2015 ITFC and 2019 HRS recommendations. Similarly, in the patient cohort from *Casella* et al., an ARVC risk calculator-derived 5-year risk threshold ranging between 12.5% and 17.5% was identified as superior to the 2015 ITFC algorithm.^32^ The analysis from Baudinaud *et al.* instead showed risk overestimation from the ARVC risk calculator for predicted risk estimates <50%; nonetheless, the ARVC risk calculator still outperformed the 2015 ITFC in their patient population.^53^ Finally, in the ARVC patient population presented by Jordà *et al*. for model validation, the ARVC risk calculator clinical benefit resulted superior to the 2015 ITFC, 2017 AHA, and 2019 HRS ICD placement recommendations at all given thresholds, with the ARVC risk calculator and the 2019 HRS performance becoming similar for 5-year risk estimates of ∼35%.^55^ The risk calculator seems therefore to perform better for arrhythmic risk stratification in primary prevention patients with ARVC than all the currently available risk stratification guidelines. This tool has been tested and found effective in a significant patient population (more than 1500 different ARVC patients combined) ascertained from different specialists (electrophysiologists and heart failure experts) and across different continents (Europe, America, and Asia).

One of the major unanswered questions in primary prevention of VA generally is whether specific risk thresholds should be used to guide ICD placement. Increasingly, guidelines are moving towards a more nuanced approach in which a reliable risk estimate is only one part of a discussion between patient and their healthcare team. Patient preferences and values should inform this discussion, and there are likely important gender-related, cultural and socio-economic factors that may need to be considered. Moreover, the realities of specific healthcare systems inevitably colour discussions about thresholds of ‘acceptable risk’. In this context, the ARVC risk calculator does not replace the human element in disease management^79^ but instead provides a rational, evidence-based tool that can be integrated into a comprehensive and holistic clinical workflow.

## Future directions

The current ARVC risk calculator is appropriate for patients fulfilling a definite diagnosis of ARVC. However, while gene-elusive and *PKP2* variants represent the majority of ARVC cases fulfilling 2010 TFC at the time of their first sustained VA, fewer than half of patients carrying variants in genes such as *DSP*, *PLN*, and *FLNC* do so.^7,80–82^ Patients with these genotypes represent a distinct ACM subpopulation, with biventricular and left-dominant phenotypes significantly differing from the classical RV dominant disease for which ARVC guidelines were developed. While these genotypes are associated with a significant arrhythmic burden, the most appropriate risk stratification strategies for these patients remain an active area of investigation. Analyses from Casella *et al*.^32^ and Aquaro *et al*.^41^ reported a significant underprediction ARVC risk calculator-derived VA risk in patients with a left-dominant ARVC phenotype, while Protonotarios *et al*.^26^ showed the ARVC risk calculator over-predicting arrhythmic risk in patients with a P/LP variants in the *DSP* gene fulfilling the conditions for ARVC risk calculator usage.

The recognition that the presentation and natural history of heart muscle diseases are heavily influenced by common and rare genetic variation is propelling efforts to evolve the current phenotype-based approach to diagnosis and risk stratification to one based on a more comprehensive disease description that includes genotypical aetiology.^48^ Among patients with a 2010 TFC phenotype, Protonotarios *et al*.^26^ clearly showed the strong importance of the underlying genotype when assessing individual ARVC patients’ risk for VA. A recent study from Paldino *et al*. showed that a genotype-based classification of cardiomyopathies allows an improved long-term arrhythmic outcome stratification compared with a phenotype-based one among patients with genetically determined dilated cardiomyopathy and ARVC phenotypes.^82^ In their cohort, patients with *DSP*, *LMNA*, and *FLNC* variants experienced consistent VA event rates regardless of the fulfilment of the 2010 TFC or their initial clinical diagnosis.

Clearly, more data characterizing the impact of genotype on arrhythmic risk are needed. In addition to P/LP variants in different genes demonstrating significantly different rates of arrhythmic events, variants occurring in differing regions of the same gene may produce clinically significant differences in arrhythmic risk.^83^ Given the strong apparent influence of genetic information on arrhythmic events, we envision a shift towards management strategies developed from a ‘genotype first’ perspective rather than strategies developed in patient cohorts defined by phenotype alone. Although we expect many of the same VA risk factors (i.e. NSVT and RV/LV dysfunction) to be shared across ARVC patients with different underlying genetic variants, their relative weight may vary and the role of some environmental modifiers (i.e. physical exercise) may be different. Indeed, evidence is emerging that this is true for some other cardiomyopathies as well. Gene-specific algorithms have already been proposed with good results for some ARVC genotypes,^7,82^ as well as for other genetically determined cardiomyopathies,^84^ regardless of their phenotype. A precision medicine approach accounting for the genotype and for the clinical and structural characteristics of those diseases seems to be the future of the field of ACM.

## Suggested approach to disease assessment

**Figure 3:**
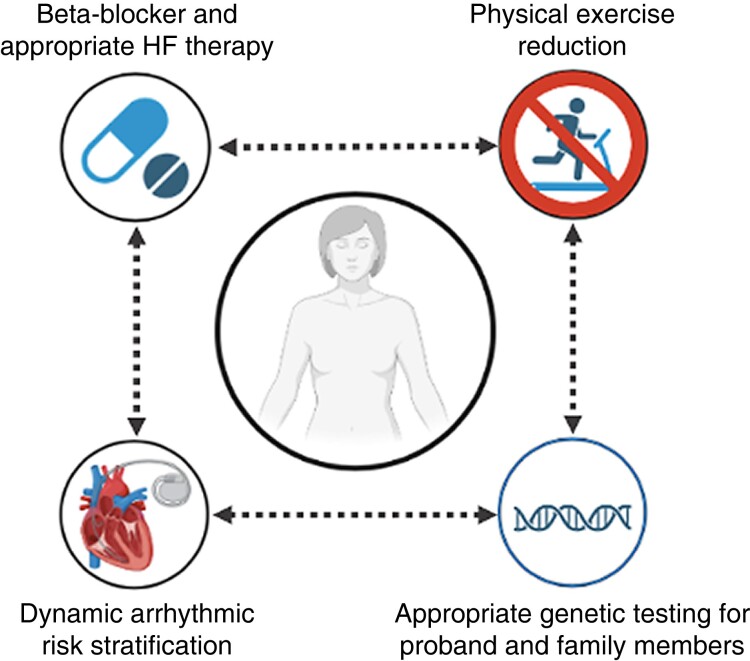
Summary of the clinical pillars for the management of patients with ARVC. HF, heart failure.

When evaluating a patient with suspected ARVC, the first task faced by a clinician is to determine if they in fact have ARVC (*Figure 3*). Currently, the 2010 Task Force Criteria are the benchmark criteria that are well accepted and have been the foundation of all the recent research studies. Nonetheless, the possibility of diagnostic overlap with other arrhythmic syndromes, cardiomyopathies, or exercise-induced adaptations is well known.^59,85–89^ Referral of patients with an unclear final diagnosis of ARVC to high-volume expert centres is reccomended, where advanced imaging labs and dedicated cardiogenetic programmes for clinical core lab may help in reaching an appropriate final diagnosis. Due to the strong importance of the underlying gene variant, genetic testing at the first patient assessment is appropriate.

Once an ARVC diagnosis is established or strongly suspected, the next priority is to estimate their individual arrhythmic risk. If a patient has had a prior sustained VA, their risk of a potentially life-threatening VA is high enough to warrant consideration of ICD implantation. For individuals without a prior episode of sustained VA, the ARVC risk calculator is a helpful and easily implemented tool that facilitates informed discussion about prophylactic ICD implantation. At this point, patient preferences and values play an important role.^90^ Some patients are very concerned about any risk of cardiac arrest and welcome the security provided by an ICD. Other patients are reluctant to consider a device despite the risks at stake. A case-by-case discussion between patient and physician should be held at the time of the first risk assessment and then at intervals during follow-up.

Patients should be counselled to avoid all competitive and endurance sports and to not exceed activity levels suggested by the ACC/AHA guidelines for a healthy lifestyle.^91^ Additionally, they should be on a beta-blocker and, if ventricular dysfunction is present, heart failure–optimized medical therapy. Anti-arrhythmic medications (i.e. flecainide) and more invasive procedures (i.e. catheter ablation for VT or other complex arrhythmias) instead, although safe and exceedingly useful for the management of some patients, at the current state of evidence should not be offered to all patients with ARVC but implemented on a case-by-case basis.^92–99^ Furthermore, they should have an ECG and Holter every year and repeat imaging with an echocardiogram and/or CMR every 2 or 3 years. These new clinical studies should be used to repeat and update the risk assessment using the ARVC risk calculator, to dynamically track changes in the predicted risk of arrhythmic events. Changes in symptoms, especially with syncope or pre-syncope, should prompt immediate re-evaluation. Finally, screening of relatives of ARVC patients to facilitate early diagnosis and to prevent SCD should be considered.^100,101^ Genetic testing can strongly inform this process. When an ARVC patient has a P/LP variant associated with their disease cascade, genetic testing in conjunction with cardiac screening is recommended. Asymptomatic family members with normal ECG and imaging who have not inherited a familial variant may be discharged from follow-up while relatives with a P/LP variant require longitudinal follow-up.^48^ At-risk first-degree relatives of gene-elusive ARVC patients should also be screened although the optimal timing is still uncertain.^102,103^

## Limitations of the calculator

The current ARVC risk calculator presents three main limitations that should be highlighted in order to provide the reviewer with a complete assessment of this tool. The first limitation regards its applicability: currently, only patients with an ARVC diagnosis as per the 2010 Task Force Criteria are eligible for its use in the clinical setting. This inclusion criterion prevents patients presenting with other forms of ACM (mainly those presenting with a left-sided disease *ab initio*) to benefit from this risk stratification strategy. With the upcoming introduction of an even more refined gene-first classification and stratification approach, we hope that gene-specific risk stratification tools will be developed in the near future, to overcome this limitation. The second limitation regards the primary endpoint predicted by the calculator, which is a composite of a combination of sustained VA and ICD therapies. While clinically meaningful, ICD shocks are but an imperfect proxy for SCD events and it is difficult to address how many of those events may have degenerated into an actual SCD event.^79^ The version of the risk calculator predicting only fast VA and SCD events^21^ is yet waiting for external validation. This point should be carefully considered before clinical decision-making is performed with this tool, which is not meant to replace but to aid individual physician expertise and inform and empower individual patients. Finally, several additional disease risk features that have been described over the years (i.e. presence of LGE in the LV, the development of ‘hot phases’ of disease/episodes of myocarditis, or the value of low potentials and scarring at electro-anatomical mapping) may be of additional value in a risk stratification strategy based on the ARVC risk calculator. Multiple studies are currently being performed to integrate the data in the risk calculator as well, and it is our hope that new, more comprehensive versions of the risk calculator will be made available in the near future.

## Conclusion

This review represents a comprehensive summary of the current state of the art in the field of risk stratification for patients with ARVC. The management of patients with ARVC and their family members is a complicated task. The progress achieved over the last few years, however, allows us to have a bright hope for the future. As our understanding of this disease will progressively increase over the upcoming years, with new additional gene-specific insights being unlocked by multiple groups across the planet, we hope that soon even more patient-specific and individual-tailored risk stratification will become available to the clinicians, with our main goal remaining the minimizing of SCD events in ARVC, while avoiding ICD implantation in subjects not likely to require ICD therapy.
